# Outbreaks of the Brown Planthopper *Nilaparvata lugens* (Stål) in the Yangtze River Delta: Immigration or Local Reproduction?

**DOI:** 10.1371/journal.pone.0088973

**Published:** 2014-02-18

**Authors:** Gao Hu, Fang Lu, Bao-Ping Zhai, Ming-Hong Lu, Wan-Cai Liu, Feng Zhu, Xiang-Wen Wu, Gui-Hua Chen, Xiao-Xi Zhang

**Affiliations:** 1 Key Laboratory of Integrated Management of Crop Diseases and Pests (Ministry of Education), College of Plant Protection, Nanjing Agricultural University, Nanjing, China; 2 Division of Pest Forecasting, China National Agro-Tec Extension and Service Center, Beijing, China; 3 Plant Protection Station of Jiangsu Province, Nanjing, China; 4 Plant Protection Station of Shanghai City, Shanghai, China; 5 Plant Protection Station of Jinhua City, Jinhua, China; USDA-Agricultural Research Service, United States of America

## Abstract

An effective control strategy for migratory pests is difficult to implement because the cause of infestation (i.e., immigration or local reproduction) is often not established. In particular, the outbreak mechanisms of the brown planthopper, *Nilaparvata lugens* (Stål), an insect causing massive losses in rice fields in the Yangtze River Delta in China, are frequently unclear. Field surveys of *N. lugens* were performed in Jiangsu and Zhejiang Provinces in 2008 to 2010 and related historical data from 2003 onwards were collected and analyzed to clarify the cause of these infestations. Results showed that outbreaks of *N. lugens* in the Yangtze River Delta were mostly associated with an extremely high increase in population. Thus, reproduction rather than immigration from distant sources were the cause of the infestations. Although mass migration occurred late in the season (late August and early September), the source areas of *N. lugens* catches in the Yangtze River Delta were mainly located in nearby areas, including the Yangtze River Delta itself, Anhui and northern Jiangxi Provinces. These regions collectively form the lower-middle reaches of the Yangtze River, and the late migration can thus be considered as an internal bioflow within one population.

## Introduction

Numerous migratory insects, such as *Schistocerca gregaria* (Forskal), *Chortoicetes terminifera* (Walker), *Locusta migratoria manilensis* (Meyen), and *Cnaphalocrocis medinalis* (Guenée), are pests to agricultural crops, and their population outbreaks often cause immense crop damage and serious economic problems [1]–[5]. The population growth of migratory insects has been attributed to migration and local reproduction. Local pest populations could grow exponentially, at least for a time, because of their high intrinsic rate of increase. In contrast, host plants could be devastated immediately after mass immigration, such as in locusts [2], [3]. Therefore, determining the intrinsic cause of pest population outbreaks under the simultaneous effect of immigration and local reproduction is difficult. Here, we were specifically concerned with determining the cause of Nilaparvata lugens (Stål) infestations in the Yangtze River Delta in order to significantly improve pest forecasting and control of this pest.

**Figure 1:**
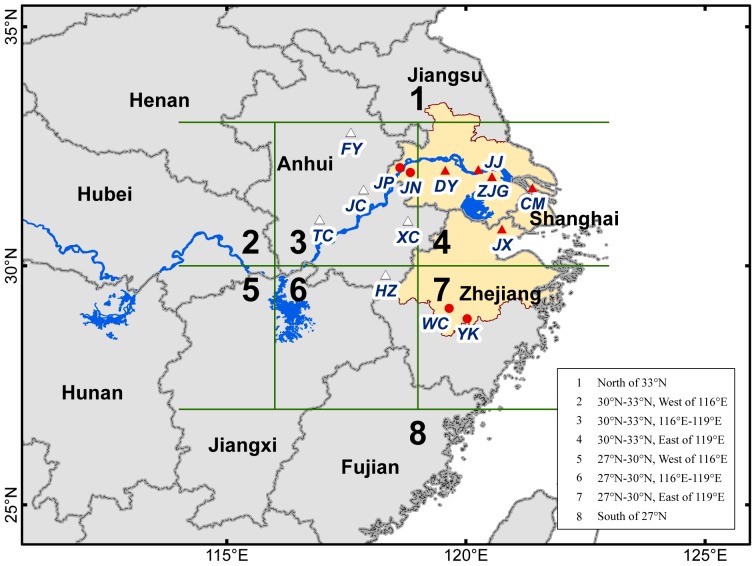
Location of experimental sites and the probable source area of *N. lugens* in the Yangtze River Delta. Notes: The region filled with yellow represents the Yangtze River Delta. Area 1 includes northern Jiangsu and northern Anhui; Area 2 includes southeastern Henan and eastern Hubei; Area 3 includes Southern and central Anhui; Area 4 includes the Yangtze River Delta; Area 5 includes northeastern Hunan and northwestern Jiangxi; Area 6 includes northeastern Jiangxi, northwestern Fujian, and western Zhejiang; Area 7 includes southern and central Zhejiang; Area 8 includes southern Jiangxi and southern and central Fujian. “•” represents the experimental sites in Nanjing and Jinhua City, JP-Jiangpu, JN-Jiangning, WC-Wucheng, and YK-Yongkong. “▴” represents stations in the Yangtze River Delta, CM-Chongming, DY-Danyang, JJ-Jingjiang, ZJG-Zhangjiagang, and JX-Jiaxing; “Δ” represents stations in the probable source areas of *N. lugens*, FY-Fengyang, JC-Juchao, TC-Tongcheng, XC-Xuancheng, and HZ-Huizhou.

*N. lugens* is one of the most serious rice pests in the temperate and tropical regions of East and Southeast Asia. Since 1968, *N. lugens* has greatly damaged the rice industry in China and in other countries in Asia [6], [7]. In the past few years, new outbreaks of *N. lugens* have been continuously recorded. For example, the loss of rice yield in China caused by *N. lugens* was approximately 1,880,000 t in 2005 [8]–[10]. The Yangtze River Delta, including Shanghai Municipality, southern Jiangsu, and northern Zhejiang Provinces (Fig. 1), is one of the most important rice-producing regions in China. The occurrences of *N. lugens* outbreaks in this region, including those in 2005 and 2006, are more severe and more frequent compared with those in other regions. The problem of *N. lugens* outbreaks in the Yangtze River Delta has elicited more attention and investment compared with other areas; thus, it is the primary focus of this paper.

*N. lugens* has an annual return migration in East Asia. *N. lugens* cannot overwinter in temperate zones, such as mainland China, Japan, and the Korean Peninsula. Instead, an infestation is initiated by windborne spring/summer migrants from the south [6], [7], [11]–[14]. Infestation begins with a northward migration that occurs in late March every year. The distribution of *N. lugens* then further expands northward by progeny of the migrant population and can cover the entire rice-growing region in China, as well as Japan and the Korean Peninsula. From September onwards, the general direction of planthopper migration becomes predominantly southbound [15], [16]. At the end of October, most *N. lugens* populations are only present in the safe overwintering areas, mainly in the Indo–China Peninsula, within which a proportion of successful return migrants is present [8], [11], [12], [14]. The immigration peaks of *N. lugens* always appear in mid- to late June and mid- to late July in the Yangtze River Delta when the early season rice ripens in the southern and northern areas of South China. *N. lugens* reproduces three generations and causes considerable damage on single-crop late-maturing rice in late September [8], [12].

The lower-middle reaches of the Yangtze River are divided into two types of cultivation based on the rice planting system used. One area comprises the Yangtze River Delta, wherein single cropping rice has been dominant since 1997.The other area, including Hubei, southern and central Anhui, northern Hunan, and northern Jiangxi, consists of mixed cultivation wherein both single and double cropping techniques are used. When *N. lugens* in South China begins to emigrate because of the decline in rice availability, the large areas of single-crop rice in mixed planting areas are suitable for their continued survival and breeding. Consequently, the population rapidly grows, which leads to a mass late migration in the Yangtze River Delta where the single-crop rice is at the heading stage during late August to early September [8]. By contrast, migration before mid-August is called early migration. Hu et al. (2011) believe that outbreaks of *N. lugens* in the Yangtze River Delta are frequent because of mass late migration caused by the increased area of the single-crop rice in the mixed planting area in the lower-middle reaches of the Yangtze River [8], [10], [17]–[19].

However, *N. lugens* can reproduce two to three generations from colonizing in June and July or until the rice reaches the yellow ripe stage in early to mid-October in the Yangtze River Delta. *N. lugens* is an “r-strategy” organism; thus, the population size of this species could become very large after two or three generations. Based on this perspective, Cheng and Zhu (2006) concluded that the high growth rate of *N. lugens* is the key to outbreaks in the lower-middle reaches of the Yangtze River, wherein the immigrants provide the initial population and the high number of *N. lugens* catches by light trap in the late season comes from the local population [20]. However, this hypothesis contradicts the fact that emigrating planthoppers (produced by the build-up of local populations) are not thought to be caught by local light traps [21]–[24].

Field surveys of the population dynamics of *N. lugens* were performed in Jiangsu Province and Zhejiang Province from July to September (2008 to 2010) to resolve the conflicting hypotheses. The data analyzed here were from 2003 to 2010 including systematic field investigation to define the important factors of the outbreak. This study aims to provide insights into ways of forecasting and controlling the population outbreaks of *N. lugens* by studying the outbreak mechanisms of this pest in the Yangtze River Delta.

## Materials and Methods

### Systematic field investigation

Systematic field investigations were conducted to explore the population dynamics of *N. lugens* in the Yangtze River Delta. In each experimental site, the one or two paddies selected were moderately fertile with routine cultural practices, and, ideally, no pesticides were used to control pests during the rice-growing season. The rice varieties in most areas of the Yangtze River Delta are the local commercial varieties of japonica rice. Systematic field investigation of *N. lugens* populations was performed once every 3 d by the plant-shaking method. A plate (39 cm×29.5 cm×2 cm) was inserted at the base of the rice plants, and *N. lugens* samples were obtained by shaking the plants. The number of *N. lugens* per hill (i.e. all tillers growing from a seedling or a “clump”) was counted manually [8].

Systematic field investigations were conducted in Nanjing City, Jiangsu Province from 2008 to 2010. Similar investigations were also carried out in Jinhua City, Zhejiang Province, since 2009 (Fig. 1). The experimental sites were set by the Plant Protection Station of each city; thus, the actual locations of the paddies were different for each year (Table 1). The selected paddies were leased from the local farmers. Farmers always tried to control the pest when no observers were present, and thus pesticides were applied in 2010 (Table 1).

**Table 1:** Information on experimental paddies from 2008 to 2010.

Locations	Survey periods	Pesticide used
Jiangning, Nanjing	2008/8/3-10/6	No
Jiangning, Nanjing	2009/7/9-9/27	No
Yongkang, Jinhua	2009/7/11-9/27	No
Jiangpu, Nanjing	2010/7/31-9/23	8/3, 8/20: pesticide was applied to control rice planthopper
Wucheng, Jinhua	2010/7/11-9/25	8/13, 9/3: pesticide was applied to control rice planthopper

According to the rules of investigation and forecast for *N. lugens* and *Sogatella furcifera* Horváth [25], the degree of occurrence was classified into the following five levels based on the number of *N. lugens* per 100 hills: light (<500), moderate (between 500 and 1000), high (between 1000 and 2000), outbreak (between 2001 and 3000), and severe outbreak (>3000).

### Ovary dissection

Macropterous females of *N. lugens* were collected from experimental paddies and dissected every 3 d to estimate the level of ovary development. Using this method, Chen et al. (1979) [26] classified the ovarian development into five levels and related it to the migration status of the population. According to Chen et al.'s criteria, the level of ovary development of the insects gathered from the source areas or during the emigration period was at level I. By contrast, the ovaries of most female insects collected at the landing areas or during the immigration periods were mature with ovary development level III or above because the oocytes underwent continuous and immediate development upon landing of the insect [8], [26], [27].

### Light trap

In each experimental site, a black light trap was placed less than 20 m away from a rice paddy. The trap consisted of a 20 W black light lamp with a top cover, set on top of a pole, 2 m above ground. These lamps were switched on at 1900 h (Beijing Time, same thereafter) and off at 0700 h next morning every day during the systematic field investigation. The catches in the light traps were collected every morning at 0900 h and were identified using a stereomicroscope. *N. lugens* catches were collected and counted every 2 h each night in Nanjing City in September 2010 to observe the nocturnal flight dynamics of the planthoppers.

### Other light trap data and field observation data

The daily *N. lugens* light trap data and 5-day field systematic survey data during 2003 to 2010 from plant protection stations of 10 counties, including Danyang (Jiangsu), Zhangjiagang (Jiangsu), Jingjiang (Jiangsu), Chongming (Shanghai), Jiaxing (Zhejiang), Huizhou (Anhui), Tongcheng (Anhui), Xuancheng (Anhui), Fengyang (Anhui), and Juchao (Anhui), were obtained by the China National Agro-Tec Extension and Service Center (Fig. 1). The five stations in the Yangtze River Delta were studied in mid- to late August and mid- to late September since 2003 to ascertain the occurrence pattern of *N. lugens* in the Yangtze River Delta.

Traditional black light traps (20 W black light lamp) were used to catch planthoppers before 2005. From 2005 onwards, the black light traps were replaced with frequoscillation lamps (20 W black light lamp, Jiaduo Brand, Jiaduo Science, Industry and Trade Co. Ltd., China). The frequoscillation lamps were switched on at 1900 h and off at 0700 h in the next morning every day at the same time from April 1 to November 15.

### Population increase rate of *N. lugens* in the Yangtze River Delta

*N. lugens* migrated from South China to the Yangtze River Delta before mid-August. Rice plants in the Yangtze River Delta were at the mature stage after mid-September. *N. lugens* mainly causes severe damage to rice crops in this region from mid-September. Thus, the population increase rate (*R*) in the late season was defined as the ratio of the total number of nymphs and adults when the number of early instar nymphs (instar I-III) peaked in mid- and late September to the corresponding peak in mid- and late August. The period between the early instar nymph peak in August and September was approximately equal to one generation duration of N. lugens. A high population increase rate was defined as a ratio of >40 [28], [29].

### Trajectory analysis

Probable source areas of migratory *N. lugens* were defined by constructing backward/forward trajectories, which were based on the following assumptions: (i) planthoppers are displaced downwind [8], [12], [15], [21], [30]–[32]; (ii) migration normally starts at dusk and partly at dawn [8, 12, 115, 21, 30–32]; (iii) migrants frequently concentrate at the height of approximately 1,000 m [15], [32]; and (iv) the planthoppers cannot fly in an atmosphere of <16.5°C [8], [15], [21], [30]. The NOAA ARL HYSPLIT model [8], [33], [34] was applied to calculate backward trajectories. The backward trajectories for light trap locations were calculated every 2 h during peak periods (1900 h to 0500 h of the next day) and terminated at the take-off time of *N. lugens* (viz. 1900 h on the day and 1900 on the day before that day), with the initial height at 500, 1,000, and 1,500 m aboveground. The duration of trajectories did not exceed 34 h [12], [35], [36]. An endpoint of a trajectory was considered a probable source if it was located in a rice planting area where the crop was at a later growth stage.

For the five stations (Chongming, Danyang, Jingjiang, Zhangjiagang, and Jiaxing), 90 peak days from August 21 to September 10 (2003 to 2010) were selected to calculate backward trajectories. The number of trajectory endpoints was counted in each 1°×1° grid to represent the source area. The whole probable source area was divided into eight zones (Fig. 1), and the probability of valid trajectory endpoints in each zone was then calculated.

### Meteorological data

Data on meteorological factors (2003 to 2010), including temperature and wind, were obtained from the China Meteorological Data Service System (http://cdc.cma.gov.cn). The temperature data were expressed as the daily mean value. Wind direction data at the height of 850 hPa were obtained from the Shanghai Municipality at 0800 and 2000 h every day.

### Statistical analysis

For a 5% rejection threshold, the association between population increase rate and size of light-trap catch in outbreak and non-outbreak years in five stations from 2003 to 2010 were tested using Chi-square analysis and obtained using *R* (version 3.0.0, http://www.r-project.org/). When sample sizes were less than 5, Fisher's exact test was then applied.

### Ethics statement

No specific permits were required for the described field studies. The brown planthopper *N. lugens* (Stål) is a major pest of rice in Asia, and huge amounts of manpower and resources are used to control the damage it causes every year.

In this study, we confirmed the following: (i) the location is not privately owned or protected and (ii) the field studies did not involve endangered or protected species.

## Results

### Population dynamics of *N. lugens* in the Yangtze River Delta

**Figure 2:**
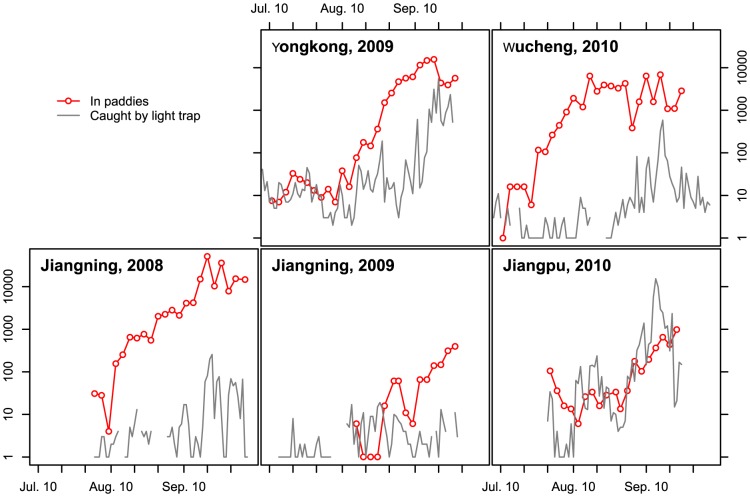
Number of *N. lugens* in paddies and caught by light trap at experimental sites.

**Figure 3:**
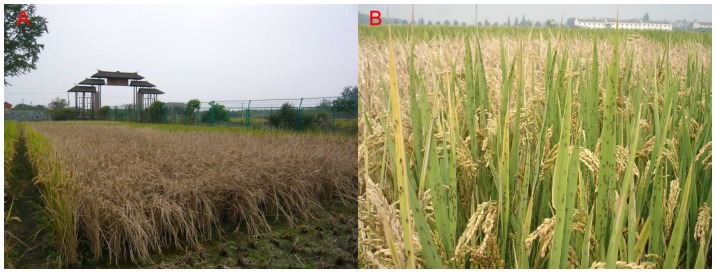
“Hopper burn” (A) and macropterous adults swarming on rice leaves (B) in Jiangning District, Nanjing City, in 2008. Note: Pictures were taken on October 2 (yellow ripe stage). Rice was harvested on October 20.

The *N. lugens* population in all experimental paddies was at a low density before late July and generally peaked in the second half of September (Fig. 2). In three out of the five paddies, the *N. lugens* population exceeded 3,000 insects per 100 hills in late September. The three paddies were in Jiangning (the maximum population was 51,140 insects per 100 hills, September 20, 2008), Yongkang (15,673 insects per 100 hills, September 18, 2009), and Wucheng (6,885 insects per 100 hills, September 16, 2010). In the first two paddies, the “hopper burn” (browning of the leaves or withering of the whole plant) areas were approximately 60% and 10% (Fig. 3 and Table 2). The damage in the paddy in Wucheng was minimal. However, excluding the paddy in Wucheng, the increase rates of *N. lugens* population in the other two paddies with high density were much greater than 40, which was defined by previous studies as a high increase rate (Table 2) [29]. In the paddy in Wucheng, the population increase rate was kept low after August 10. However, pesticide control was performed twice to suppress the increase in *N. lugens* population at this site (Tables 1 and 2).

**Table 2:** Population increase rate of investigation fields from 2008 to 2010.

Year	Station	number of *N. lugens* per 100 hills		Proliferation multiple	Area of hopper burn (%)	Catches of *N. lugens* (Aug. 21–Sep. 10)
		Aug.	Sept.			
2008	Jiangning	645 (18)*	51140 (20)	79.29	60	58
2009	Jiangning	15 (28)	395 (27)	26.33	0	110
2009	Yongkang	124 (19)	15070 (18)	121.53	10	688
2010	Jiangpu	28 (24)	980 (23)	35.00	0	3681
2010	Wucheng	6335 (17)	6885 (16)	1.09	0	177

* The numbers in the brackets represent the date when the nymphal population was in early instars (I–III). The periods between the Aug. and Sept. dates were almost one month long and were nearly equal to the generation time of N. lugens.

During the late season (after mid-August), three main peak periods of *N. lugens* light trap catches were recorded in the experimental sites. The first two periods were noted in late August and early September, i.e. before the population densities in the paddies reached the maximum value. However, whether these catches were immigrants from distant sources was unclear. Among these cases, the catches of *N. lugens* in Jiangpu in 2010 were the highest (3,681 specimens caught from August 21 to September 10) (Fig. 2 and Table 2). In the second peak periods (September 7 to September 9) in Jiangpu in 2010, 2,697 individuals were caught in these three nights. Up to 1,397 out of these 2,697 individuals (48.20%) were caught during 0200 h to 0500 h, and 1,114 (41.31%) were caught around midnight (2100 h to 0200 h). The individuals caught by light trap at midnight and later must have been immigrants.

In each experimental paddy, >100 macropterous females were collected and dissected during the first two peak periods of light trap catches, viz. in late August and early September. Excluding 2010, the individuals with ovaries at levels I and II accounted for >70% of the entire population, whereas those at level III and above accounted for <30% (Table 3). According to the classification criteria established by Chen et al. (1979), these values characterize an emigrating population. Thus, the macropterous adults were from relatively local populations, and the high increase rates were attributed to reproduction rather than immigration. In 2010, the individuals with ovaries at level III and above accounted for a distinctly higher proportion of the population (>30% but <60%) (Table 3). Thus, according to the classification criteria established by Chen et al. (1979) [26], [27], the population consisted of local insects that continued to breed in approximately the same area or that mixed with immigrants.

**Table 3:** Ovarian development of *N. lugens* in the experimental paddies from 2008 to 2010.

Year	Time	Station	Number of *N. lugens* dissected	Grade of ovarian development		
				Level I	Level II	≥ Level III
2008	8/23**–**9/11	Jiangning	186	168 (90.32%)	9 (4.84%)	9 (4.84%)
2009	9/9**–**9/18	Jiangning	111	35 (31.53%)	73 (65.77%)	3 (2.70%)
2009	8/19**–**9/12	Yongkang	106	68 (64.15%)	8 (7.55%)	30 (28.30%)
2010	9/9**–**9/16	Jiangpu	154	33 (21.43%)	47 (30.52%)	74 (48.05%)
2010	8/20**–**9/10	Wucheng	195	120 (61.54%)	4 (2.05%)	71 (36.41%)

These results showed that the population growth of *N. lugens* in the late season in the Yangtze River Delta was evidently caused by reproduction rather than immigration in 2008 and 2009. The outbreaks in Jiangning (2008) and Yongkong (2009) resulted from the high increase rate of the local population. In 2010, numerous immigrants maintained the large population, whereas the local population was suppressed by pesticide control.

### Relationship between autumn temperature and outbreaks of *N. lugens* in the late season

The temperature in Jinhua was consistently higher than in Nanjing (Table 4). Accordingly, the population sizes of *N. lugens* were larger, and the population peaks were earlier in Jinhua (Table 2). Excluding Nanjing in 2009, the temperatures were near or above 26°C before the population densities in the paddies reached the maximum value and were suitable for the population growth of *N. lugens* (Table 4). In Nanjing in mid-September of 2009, the temperature was only 22.8°C and the *N. lugens* population may have been suppressed by these cool temperatures. The population of *N. lugens* in 2010 was controlled by pesticides, so whether a suitable temperature simulated the increase in *N. lugens* population in this season is unclear.

**Table 4:** Average temperature from August 21 to September 30 every 10 d during 2008 to 2010 (°C).

Year	Nanjing				Jinhua			
	8/21–8/31	9/1–9/10	9/11–9/20	9/21–9/30	8/21–8/31	9/1–9/10	9/11–9/20	9/21–9/30
2008	25.5	24.4	25.7	23.0	-	-	-	-
2009	26.8	25.2	22.8	22.3	29.7	28.0	26.5	24.5
2010	26.7	27.1	25.5	20.1	29.3	29.2	27.9	21.4
Mean*	27.0	25.2	24.2	22.3	28.7	26.8	25.7	24.0

* Mean value for 2000–2010

### Occurrence of *N. lugens* in the Yangtze River Delta from 2003 to 2010

According to the field survey data in 2003 to 2010, most stations in the Yangtze River Delta suffered outbreaks in 2005, 2006, and 2007, whereas occurrences were light in other years. In the five stations (Chongming, Danyang, Jingjiang, Zhangjiagang, and Jiaxing) during these eight years, 14 out of 40 cases (35.0%) reached the outbreak level, viz. the number of *N. lugens* was >3,000 per 100 hills (Table 5).

**Table 5:** Number of *N. lugens* per 100 hills and population increase rate in the Yangtze River Delta from 2003–2010.

Station	Month	Number of *N. lugens*							
		2003	2004	2005	2006	2007	2008	2009	2010
Chongming	Aug.	10	28	85	38	30	12	8	86
		(20) §	(20)	(20)	(25)	(25)	(20)	(20)	(20)
	Sept.	319	101	357	24030	1315	291	49	622
		(20)	(20)	(20)	(20)	(20)	(15)	(15)	(15)
	R*	31.90	3.61	4.20	632.37	43.83	24.25	6.13	7.23
Danyang	Aug.	12	56	72	1797	162	170	264	54
		(20)	(15)	(25)	(20)	(20)	(31)	(31)	(31)
	Sept.	114	56	1230	6505	735	180	254	1404
		(20)	(15)	(30)	(20)	(15)	(25)	(25)	(25)
	R	9.50	1.00	17.08	3.62	4.54	1.06	0.96	26.00
Jingjiang	Aug.	42	10	318	1043	138	147	112	45
		(20)	(20)	(25)	(25)	(25)	(25)	(25)	(15)
	Sept.	30	18	21895	12348	10862	10671	5046	5298
		(20)	(25)	(20)	(25)	(25)	(25)	(20)	(20)
	R	0.71	1.80	68.85	11.84	78.71	72.59	45.05	117.73
Zhangjiagang	Aug.	46	108	210	151	13	87	54	5
		(25)	(25)	(25)	(15)	(15)	(20)	(15)	(25)
	Sept.	640	1106	965	4678	54	73	10	10
		(25)	(25)	(20)	(25)	(15)	(25)	(15)	(25)
	R	13.91	10.24	4.60	30.98	4.15	0.84	0.19	2.00
Jiaxing	Aug.	37	37	1	1891	100	13	7	186
		(15)	(20)	(20)	(20)	(20)	(20)	(15)	(25)
	Sept.	414	293	5098	17633	4377	3812	756	8242
		(20)	(20)	(20)	(20)	(15)	(25)	(15)	(25)
	R	11.19	7.92	5098.00	9.32	43.77	293.23	108.00	44.31

* R- Proliferation multiple

§ The numbers in the brackets represent the date when the nymphal population was in early instars (I–III). The periods between the Aug. and Sept. dates were almost one month long and were nearly equal to the generation time of N. lugens.

The population increase rates of *N. lugens* in these five stations were calculated for each year (Table 5). Among the 14 outbreak cases, 10 (71.4%) occurred when population increased with a high increase rate (*R*>40), whereas only four cases (28.6%) occurred with a low increase rate (Table 6). The results of Chi-square test (*χ*2 = 14.70, *p*<0.001; Fisher's exact test, *p*<0.001) indicated that the outbreak of *N. lugens* in the Yangtze River Delta was significantly associated with the population increase rate.

**Table 6:** Frequency table comparing population increase rate and catch size for outbreak and non-outbreak situations.

	Outbreak	Non-outbreak	Total
High R and lots of catches in light trap	5	1	6
High R and few catches	5	1	6
Low R and lots of catches	4	4	8
Low R and few catches	0	20	20
Total	14	26	40

Note: R- Population increase rate

**Figure 4:**
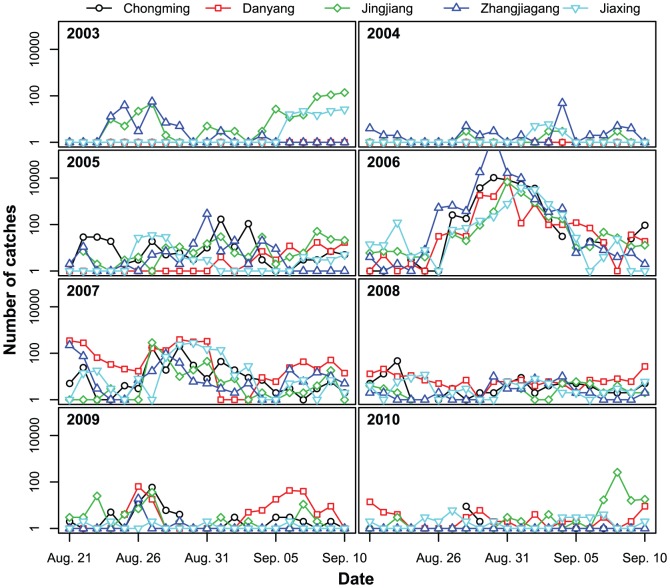
Daily catches of *N. lugens* from August 21 to September 10 from 2003 to2010.

In the five stations, the cumulative light-trap catches of *N. lugens* in early migrations were <1,000. However, the numbers in the cumulative catches during the late season varied over a wide range (Fig. 4). In the outbreak years (2005, 2006, and 2007), mass migrations occurred during late August to early September. In 2006, the cumulative catches in the late season were the highest ever recorded at several stations, with 10,000 catches per night in peak days. The maximum catch in 2006 was 790,000, which was recorded in Zhangjiagang on August 30. In the light occurrence years, the cumulative catches in the late migration were consistently minimal. In 2003 and 2009, the catches on peak days were tens, while very few rice planthoppers were caught in the late season of 2004, 2008, and 2010 (Fig. 4). Among the 14 outbreak cases, nine cases (64.29%) occurred with numerous catches in the late season, and five cases (35.71%) occurred with a few catches (Table 6). The results of Chi-square test (*χ*2 = 6.26, *p* = 0.012; Fisher's exact test, *p* = 0.007) indicated that the outbreak of *N. lugens* in the Yangtze River Delta was significantly associated with light trap catches in the late season.

Among the 14 outbreak cases, five cases occurred with both high population increase rate and numerous catches in the late season, five cases occurred with only a high population increase rate, and the other four cases occurred with only numerous catches (Table 6). These five cases with only the high population increase rate occurred from 2008 to 2009 when the occurrence level of *N. lugens* was light in the whole Yangtze River Delta, and few migrants were noted during that period. Thus, the high increase rate in these five cases was confirmed to be mainly caused by reproduction. Thus, to summarize, high reproductive rates in local or relatively local populations seemed to be the most important factors giving rise to outbreaks.

### Source areas of light trap catches in the Yangtze River Delta in the late season

Up to 1620 backward trajectories were calculated for 90 peak days in five stations, and 2,970 endpoints were obtained. After elimination of invalid endpoints (i.e. ones not ending in suitable rice cultivations), the number of available endpoints was 1,802, which accounted for 60.7% of the original 2,970.

**Figure 5:**
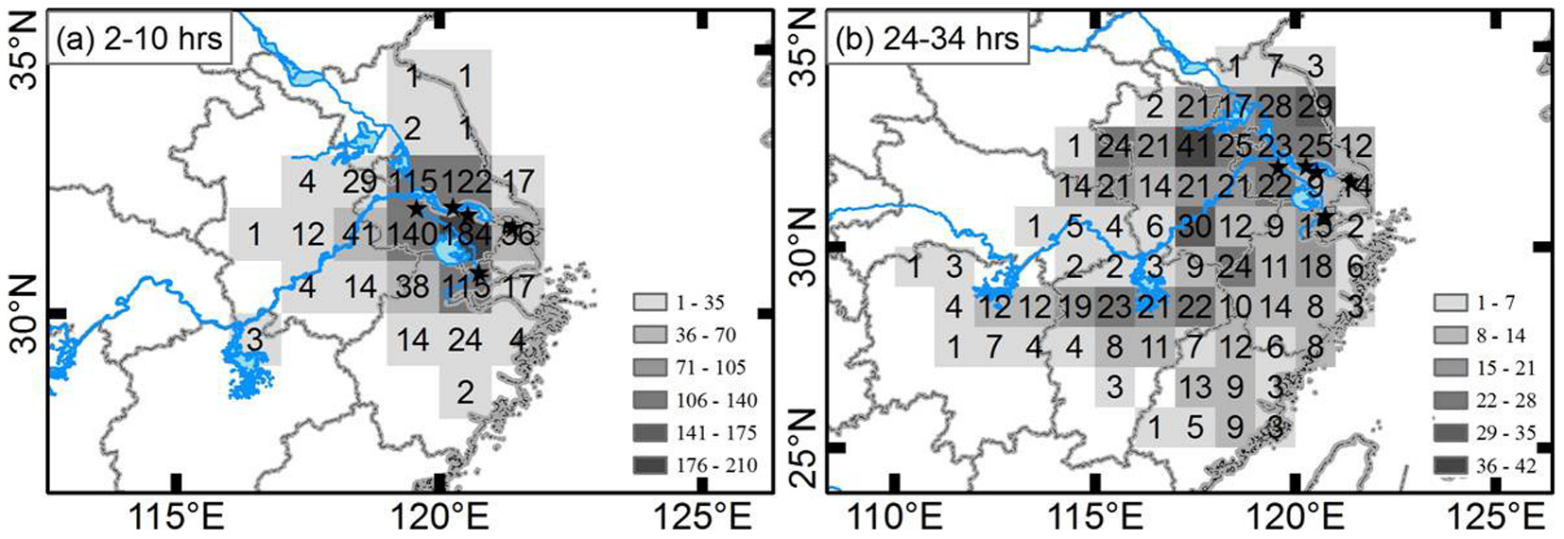
Probability distribution of endpoints of backward trajectories from Chongming, Danyang, Jingjiang, Jiaxing, and Zhangjiagang.

The source areas within 2 h to 10 h durations were still in the Yangtze River Delta (83.66% of the total), followed by southern and central Anhui (10.93% of the total). The source areas within 24 h to 34 h durations were mainly in southern and central Anhui (22.71% of the total), the Yangtze River Delta (15.58%), and northern Jiangxi (14.15%). Moreover, only a few endpoints were located in northern Jiangsu, southern Zhejiang, northern Fujian, northeastern Hunan, eastern Hubei, etc. (Table 7, Fig. 5).

**Table 7:** Probability distribution of endpoints of backward trajectories from Chongming, Danyang, Jingjiang, Jiaxing, and Zhangjiagang in different available source areas.

Year	Area	2 h–10 h		24 h–34 h	
		Amount	Probability (%)	Amount	Probability (%)
All five years	1	5	0.52	108	12.84
	2	0	0	70	8.32
	3	105	10.93	191	22.71
	4	804	83.66	131	15.58
	5	0	0	102	12.13
	6	3	0.31	119	14.15
	7	44	4.58	74	8.80
	8	0	0	46	5.47
	subtotal	961	100	841	100
Light occurrence (2003 and 2009)	1	0	0	3	1.51
	2	0	0	22	11.06
	3	51	24.29	42	21.11
	4	155	73.81	21	10.55
	5	0	0	59	29.65
	6	3	1.43	29	14.57
	7	1	0.48	22	11.06
	8	0	0	1	0.50
	subtotal	210	100	199	100
Outbreak (2005, 2006, and 2007)	1	5	0.67	105	16.36
	2	0	0	48	7.48
	3	54	7.19	149	23.21
	4	649	86.42	110	17.13
	5	0	0	43	6.70
	6	0	0	90	14.02
	7	43	5.73	52	8.10
	8	0	0	45	7.01
	subtotal	751	100	642	100

Notes: Area 1 includes northern Jiangsu and northern Anhui; Area 2 includes southeastern Henan and eastern Hubei; Area 3 includes Southern and central Anhui; Area 4 includes the Yangtze River Delta; Area 5 includes northeastern Hunan and northwestern Jiangxi; Area 6 includes northeastern Jiangxi, northwestern Fujian, and western Zhejiang; Area 7 includes southern and central Zhejiang; Area 8 includes southern Jiangxi and southern and central Fujian. These probable source area were showed in Fig. 1.

Whether the occurrence was light or severe, the source areas within 2 h to 10 h durations were therefore mainly in the surrounding area and partly in southern-central Anhui. The source areas in the Yangtze River Delta and southern-central Anhui accounted for 73.81% and 24.29%, respectively, in the years with light occurrence (2003 and 2009), and 86.42% and 7.19% in the years with outbreaks (2005, 2006, and 2007) (Table 7).

The source areas within 24 h to 34 h durations were different in the years with light occurrence compared to those with outbreaks. The endpoints during the light occurrence years were mostly in southwestern and western regions, including southern and central Anhui, northern Jiangxi, northeastern Hunan, northwestern Fujian, and western Zhejiang (65.33% of the total) (Table 7). The endpoints during the outbreak years were mostly distributed in southern and central Anhui, the Yangtze River Delta, northern Jiangsu, and northeastern Jiangxi (70.72% in total) (Table 7). Altogether, the source areas of *N. lugens* within 24 h to 34 h durations across all the years were mainly in Anhui and northern Jiangxi, which were located west of the Yangtze River Delta.

**Figure 6:**
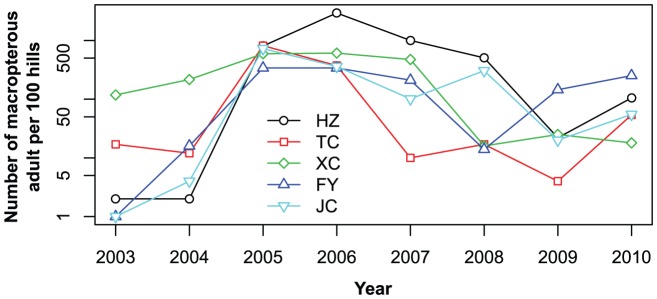
Peak number of *N. lugens* macropterous adults in Anhui Province from August 21 to September 10 from 2003 to 2010. Note: HZ-Huizhou, TC-Tongcheng, XC-Xuancheng, FY-Fengyang, JC-Juchao.

The interannual variation of macropterous adult number in Anhui corresponded with the interannual variation of catches in the Yangtze River Delta. For example, in the five locations (Huizhou, Tongcheng, Xuancheng, Fengyang, and Juchao) in Anhui, the numbers of macropterous adults from August 21 to September 10 were high in 2005 to 2007, when numerous catches were also obtained in the Yangtze River Delta (Fig. 6). Thus, Anhui could be the main source area of *N. lugens* in the Yangtze River Delta.

### Wind directions in the late season in Yangtze River Delta

**Figure 7:**
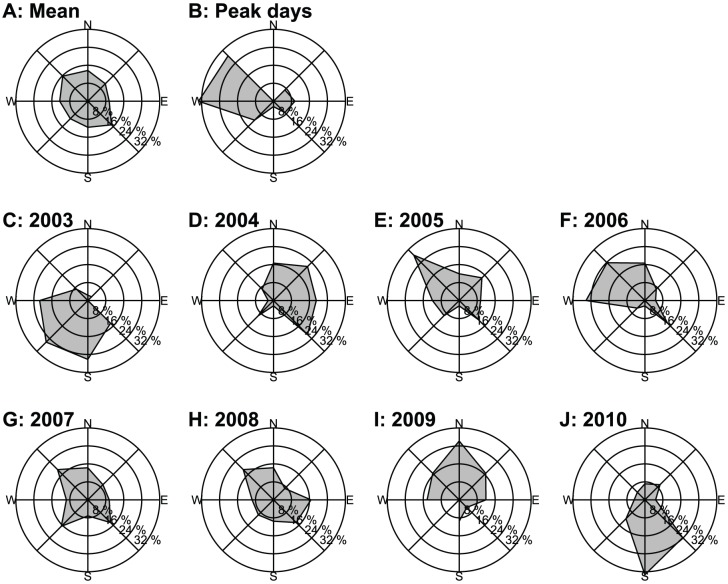
Frequency of wind direction at 850 hPa in Shanghai from 2003 to 2010. Note: (A) Mean value for 2003 to 2010, (B) mean value for peak days of *N. lugens* catches and (C-J) individual value for each year

The summer (southwest) monsoon is replaced by the winter (north-east) monsoon in eastern Asia in late August and early September. In the Yangtze River Delta, the northeasterlies are gradually established at this time. However, the winds change to southwesterly/westerly under the influence of oscillation in subtropical anticyclones. For example, in Shanghai, the wind direction at 850 hPa was mainly southeast (17.6%), southwest (17.6%), and west (18.2%) in late August and was mainly northwest (20%), north (18.8%), and northeast (18.8%) in early September. During late August to early September, the probabilities of all wind directions were similar from 2003 to 2010 (Fig. 7A). However, more west or northwest winds were noted on the 21 peak days of *N. lugens* immigration from August 21 to September 10 (2003 to 2010), with 33.3% (westerly) and 28.6% (northwesterly) probabilities (Fig. 7B). The results of Chi-square analysis revealed a significant difference in wind direction between migration days and other periods (χ2 = 24.91, p<0.001) (Table 8). Therefore, the late migration occurred more frequently under winds from the west or northwest. Considering individual seasons, during apparent late migrations in the serious outbreak years of 2005, 2006, and 2007, the frequency of wind directions was mainly west and northwest from August 21 to September 10 (Figs. 7C to 7J).

**Table 8:** Frequency of wind direction from August 21 to September 10 from 2003 to 2010 in Shanghai.

	On peak days	On nonpeak days	Total
West and northwest wind	26	69	95
Other direction Wind	16	225	241
Total	42	294	336

## Discussion

Numerous *N. lugens* were caught by light trap in the Yangtze River Delta in the late season, but the source of these planthopper catches has not exactly been identified in previous studies. Thus, it was controversial whether “late migration” occurred, and the cause of *N. lugens* outbreak (i.e., immigration or local reproduction) has also remained unclear. Similar to some previous studies, the present study confirmed that late migration was an actual phenomenon. The four main findings of this study are the following. First, many catches in the light trap occurred at or after midnight. The flight of these individuals lasted at least 6 hours after their dusk take-off [8], [12], [15], [21], [30]–[32], and thus they must be immigrants. Second, the population increase rates in the field were, in many cases, near or even greater than the maximum fecundity of *N. lugens* (705 eggs per female [37]) observed in some laboratory cases, such as *R* = 5,098 in Jiaxing in 2005 and *R* = 632 in Chongming in 2006. Third, ovary dissection verified that the macropterous females in Nanhui, Shanghai City in 2007 were immigrants (see also [8], [19]). Finally, the size of planthopper catches was related to the wind direction. Catches increased with westerly winds; thus, the major sources of the migrant insects were from the west. However, the backward trajectories in this study showed that the source areas of catches in the Yangtze River Delta in the late season were mainly located in relatively local areas, including the Yangtze River Delta itself, Anhui, and northern Jiangxi. These regions were located at a similar latitude (approximately 30°N), and the migration distances were consistently short by rice planthopper standards (mostly <300 km). When mass late migration occurred, the number of *N. lugens* in the Yangtze River Delta was consistently large, as well as that in Anhui, northern Jiangxi, and other regions at a similar latitude in eastern China. Therefore, these regions could be collectively called the lower-middle reaches of the Yangtze River. The initial population of *N. lugens* in these regions all migrated from South China in June and July. Thus, the late migration can be regarded as merely an internal flow within one population.

In the lower-middle reaches of the Yangtze River, some differences were found between the Delta itself and other areas. In the Yangtze River Delta, single-crop late-maturing rice was mostly planted, which is transplanted in mid- and late June and harvested in late October [8], [18], [19]. In other regions, including Hubei, southern and central Anhui, northern Hunan, and northern Jiangxi, the single-crop rice was subsequently transplanted between late May and early June and then matured in late August, with most of the rice harvested in early and mid-September [8], [18], [19]. These regions are closer to South China than the Yangtze River Delta, and immigrants in June and July were greater because of shorter flight distance for the insects. Moreover, the single-crop rice plants in these regions were at the tillering to booting stage and fit for *N. lugens* whose populations were thus able to grow rapidly, whereas the rice plants in the Yangtze River Delta were still immature. Furthermore, the harvest time of rice in these regions was almost one month earlier than that in the Yangtze River Delta. Thus, large numbers *N. lugens* adults emigrated as the single-crop rice was harvested in these regions, and these insects could invade the Yangtze River Delta where the rice plants were at the heading stage and now suitable for *N. lugens*. Therefore, the late migration would exacerbate the outbreak of *N. lugens* in the Yangtze River Delta, and vigilance would need to be maintained. In particular, even when the *N. lugens* population in the Yangtze River Delta was suppressed by effective control strategy, mass late migration would negate the results of control efforts.

Our study showed that outbreaks of *N. lugens* mainly occurred along with abnormally high intrinsic rates of increase. Considering 17 outbreak cases (three cases in survey data and 14 cases in historical data), 12 (70.56%) occurred with high increase rates. In other cases, where the rate of increase was low, the number of *N. lugens* was large before late August. Therefore, these outbreaks of *N. lugens* could be divided into two types. One type is called the cardinal type, wherein the number of immigrants in the early season (before late August) is unusually high, thereby causing an early dense population. Thus, this type is also commonly known as the high-immigration type [8], [11], [38]. In the historical data used for this study, the four cases with low increase rate all occurred in 2006, and the outbreak in this year was previously analyzed as a typical case of high-immigration type [38]. In the other types of *N. lugens* outbreak, called the climate type in previous studies [8], [11], [38], the population was low in the early season but increased rapidly due, in general, to meteorological factors, especially temperature. In the present study, the high increase rate was mainly attributed to reproduction rather than immigration. Although mass migration was observed in the late season, the source areas were mainly located in relatively nearby areas and the late migration can be regarded as an internal flow within one population. Thus, the other cases in this study could be classified as climate type. However, a complex type could also be present, where a relatively high early immigration and favorable climatic conditions are both contributory factors [8], [11], [38].

Temperature could control the population of *N. lugens* by direct effects on survival, reproduction, and foraging. These effects are the most crucial factors for *N. lugens* population dynamics [37], [39]. The size of the *N. lugens* population in the Yangtze River Delta was affected by temperature, as shown in the analysis of the relationship between the population dynamics of the planthopper and temperature from August to September (2008 to 2010). In the lower-middle reaches of the Yangtze River, a “cooler summer and warmer autumn” is regarded as the favored climatic condition for outbreaks of *N. lugens* [40]–[42], and this occurred for example in 2005 [8]. The occurrence of *N. lugens* in the Yangtze River Delta in 2005 was the most severe in the past 20 years [9], [10]. Hu et al. [8] suggested that steadily warmer autumns have occurred from the 1990s in the lower-middle reaches of the Yangtze River Delta and that such conditions gradually became the norm. In this study, outbreaks of *N. lugens* in the Yangtze River Delta were mainly attributed to the high reproduction rate, thus providing further evidence on why the problems caused by *N. lugens* have become more severe.

*N. lugens* population can be controlled by pesticide. Limited damage was caused by *N. lugens* in the 1990s due to the use of buprofezin and imidacloprid [11], and the high level of resistance to imidacloprid resulted in chemical control failure and great yield loss in 2005 [43]. Rive cultivars with resistance to *N. lugens* can also suppress this pest significantly [6], [44]. But, just as in the case of development of resistance to pesticide, this pest often adapted to resistant cultivars in a short time. The pest adapted to IR26, the first high-yielding cultivar with BPH resistance (with *Bph1* gene), within 2 or 3 years after being released to farmers [6]. High-yielding indica hybrid cultivars without the new resistant gene has promoted the population growth of *N. lugens* in southern China, especially in Hunan Province [45]. Therefore frequent outbreaks of *N. lugens* are not only related to suitable weather conditions, but also with other factors.

In summary, the outbreak mechanisms of *N. lugens* in the Yangtze River Delta were explored by surveying and evaluating historical data, and by determining the sources of catches in light traps in the late season. Although these catches supported the occurrence of migration, the source areas were found to be relatively close at hand and could be collectively considered, for control purposes, with sources in the Yangtze River Delta itself. Therefore, reproduction was the key factor contributing to the high population increase rate of N. lugens, and to the consequent outbreaks in this area of China. These results resolved the conflicting hypotheses on the source of these outbreak populations and will facilitate general control strategies of *N. lugens* in China.
